# Visceral Leishmaniasis in Traveler to Guyana Caused by *Leishmania siamensis*, London, UK

**DOI:** 10.3201/eid2408.180192

**Published:** 2018-08

**Authors:** Saovanee Leelayoova, Suradej Siripattanapipong, Mathirut Mungthin

**Affiliations:** Phramongkutklao College of Medicine, Bangkok, Thailand (S. Leelayoova, M. Mungthin);; Mahidol University, Bangkok (S. Siripattanapipong)

**Keywords:** visceral leishmaniasis, Leishmania siamensis, Leishmania martiniquensis, parasites, traveler, Guyana, London, UK

**To the Editor:** Polley et al. reported a case of *Leishmania siamensis* infection outside Thailand (*1*). In Thailand, 2 *Leishmania* species, *L. siamensis* (MON-324, World Health Organization code MHOM/TH/2010/TR) and *L. martiniquensis* (MON-229, World Health Organization codes MHOM/TH/2011/PG and MHOM/MQ/92/MAR1), are sporadically reported in immunocompetent and immunocompromised patients and cause cutaneous and visceral leishmaniasis (*2*). Cases of asymptomatic visceral leishmaniasis caused by both species were also detected in HIV-infected patients in Thailand (*3*).

Before 2017, *L. siamensis* was described as having 2 lineages: PG and TR. Additional information from zymodeme and genetic analysis indicated that these 2 lineages are different species (i.e., lineage PG is *L. martiniquensis* and lineage TR is *L. siamensis*) (*2*). A review of leishmaniasis cases in Thailand during 1999–2016 (*2*) summarized the biological characteristics of *L. martiniquensis* and *L. siamensis* and clarified *Leishmania* species reported in humans (Thailand and Myanmar), animals (Thailand, Germany, Switzerland, and the United States), and sand flies (Thailand).

Polley et al. (*1*) reported phylogenetic analysis of internal transcribed spacer 1 sequences of 8 isolates of *L. siamensis* (GenBank accession nos. EF200012, JX195637, GQ281279, GQ226034, JQ866907, JQ617283, JQ001751, and GQ293226) against reference sequences of other *Leishmania* species. Their results confirmed that these sequences clustered with *L. siamensis* sequences as a monophyletic group, supported by bootstrap values of 100%.

However, 7 of these sequences (GenBank accession nos. EF200012, JX195637, GQ281279, GQ226034, JQ866907, JQ001751, and JQ617283) are *L. martiniquensis* sequences (MON-229), as reported in our article (*2*). Thus, we have revised and updated our sequences submitted to GenBank regarding the species of *L. martiniquensis* (MON-229) and *L. siamensis* (MON-324) for future analysis.

The patient had a history of traveling to Caribbean Grenada, which is in the same geographic area where *L. martiniquensis* was first reported (*4*). Thus, we believe that the correct diagnosis for the 65-year-old woman in the study by Polley et al. (*1*) was visceral leishmaniasis caused by infection with *L. martiniquensis*.
